# Activity dynamics and regulation mechanism of extracellular proteases in *Bacillus velezensis* SW5

**DOI:** 10.1128/aem.01294-25

**Published:** 2025-10-14

**Authors:** Xuejin Feng, Yang Liu, Ruolin Cheng, Min Jin

**Affiliations:** 1 State Key Laboratory Breeding Base of Marine Genetic Resource, Third Institute of Oceanography, Ministry of Natural Resources 118477, Xiamen, China; 2 Fujian Key Laboratory of Agro-Products Quality and Safety, Institute of Agricultural Quality Standards and Testing Technology, Fujian Academy of Agricultural Sciences 107629, Fuzhou, China; 3 Fujian Key Laboratory of Special Aquatic Formula Feed, Fuzhou, China; Washington University in St. Louis, St. Louis, Missouri, USA

**Keywords:** *Bacillus velezensis*, transcriptome, extracellular protease, regulation mechanism

## Abstract

*Bacillus velezensis* is capable of producing and secreting a rich variety of proteases, making it an excellent catalyst for food fermentation and bioremediation, as well as a promising source of proteases with industrial application potential. However, our knowledge of the activity and regulation mechanism of extracellular proteases in *B. velezensis* during growth remains limited. In our study, we investigated the growth dynamics, extracellular protease activity, and transcriptional profiles of *B. velezensis* strain SW5 over the course of the incubation period. The growth dynamics analysis showed that the cell density of SW5 increased exponentially until 10 h post-incubation, then declined or remained stable afterward. However, the extracellular protease activity followed the opposite trend: it remained low during exponential growth but significantly increased during the stationary phase, peaking at 54 h. Transcriptomic analyses showed that key extracellular proteases, such as AprE (subtilisin), exhibited maximal expression levels at 54 h and contributed primarily to extracellular protease activity. The transcriptomic data also identified key regulatory systems of extracellular proteases, including DegQ, DegU, ComQXPA quorum-sensing system, and negative regulators (e.g., AbrB, Hpr/ScoC, and CodY), which collectively regulate protease gene expression in *B. velezensis*. Overall, our study offers comprehensive insights into the activity dynamics and potential regulatory mechanisms of extracellular proteases in *B. velezensis*, providing a theoretical foundation for the industrial application of *B. velezensis* and its proteases.

**IMPORTANCE:**

*Bacillus velezensis* holds significant economic relevance for fermentation products due to its excellent biosafety profile and strong proteolytic capabilities. Therefore, understanding the dynamics of extracellular protease activity and their corresponding regulatory mechanisms in *B. velezensis* is of great significance. In our study, we monitored the extracellular protease activity of strain SW5 and highlighted changes in the transcription levels of extracellular protease genes and their regulators throughout the incubation period. Notably, the transcriptomic data suggested that the extracellular protease activity was likely regulated by a complex network involving the DegS-DegU two-component system, the ComQXPA quorum-sensing system, DegQ, and negative regulators. Our results also revealed distinct expression patterns among extracellular protease genes and their regulators at different growth stages, highlighting the growth stage-specific regulation mode of extracellular proteases. These findings offer valuable insights into the proteolytic characteristics in *B. velezensis,* as well as the industrial application potential of *B. velezensis*.

## INTRODUCTION

*Bacillus velezensis* is an endospore-producing, gram-positive bacterium (1). It is well regarded for its metabolic versatility, resilience to extreme environmental conditions, and widespread distribution across a diversity of habitats (1–3). As of November 2024, genomic data from over 1,000 strains of *B. velezensis* were available through the National Center for Biotechnology Information (NCBI) databases, with even more genomes on the way. Like other *Bacillus* species, *B. velezensis* exhibits excellent biosafety and the ability to produce antimicrobial substances and secretory enzymes, such as proteases, lipases, and amylases (3, 4). For instance, *B. velezensis* Y1 has been shown to produce both β-glucanase and protease, with production regulated by temperature, inoculation time, pH, and inoculated liquid volume, thereby highlighting its potential for producing high protease yields under industrial fermentation conditions (5). Furthermore, this species has the potential as a plant growth-promoting agent with probiotic properties, significantly contributing to plant defense against pathogens and enhancing overall plant health (6).

In *Bacillus subtilis*, the production of extracellular proteases occurs during the stationary phase, influenced by environmental nitrogen or carbon sources and regulatory mechanisms governing gene expression (7–9). The regulatory mechanisms for extracellular protease production in *B. subtilis* are relatively well understood, primarily involving regulators (e.g., Spo0A and AbrB), the ComQXPA quorum-sensing system, DegQ, and the DegS-DegU two-component system (10, 11). Spo0A serves as a critical global regulator, directly modulating multiple genes, including the extracellular protease gene *aprE* (subtilisin), through multi-site phosphorylation (11). Spo0A can also inhibit AbrB, a negative regulator of extracellular proteases, thereby indirectly affecting extracellular protease expression (12, 13). In the ComQXPA system, ComX is modified by ComQ, which, in turn, phosphorylates ComP (sensor histidine kinase). The phosphate is then transferred from ComP to ComA, which is a regulator that binds to DNA upon phosphorylation and activates the *ComQXPA* operon (12, 13). The activation of the ComQXPA system regulates the activation of DegQ (14), which, in turn, phosphorylates DegU (14). Both activated DegQ and phosphorylated DegU can enhance the expression of extracellular proteases (14, 15). These advances in our understanding of the regulatory mechanisms controlling extracellular protease production contribute to the characterization and application of *Bacillus* proteases, particularly in industrial microbial fermentation. However, despite extensive studies on the activity and regulation mechanism of extracellular proteases in species like *Bacillus subtilis* (16–18) and *Bacillus thuringiensis* (19), our knowledge on the dynamics of extracellular protease activity and their corresponding regulatory mechanisms in *B. velezensis* remains limited, which severely hinders the industrial application of *B. velezensis*.

*B. velezensis* SW5 was isolated from fish sauce and may have evolved specialized proteolytic machinery adapted to its fish sauce fermentation niche (20). The genome of *B. velezensis* SW5 encodes various extracellular protease genes, demonstrating potentially efficient protein degradation capabilities. Recent studies identified 12 proteases, such as subtilisin E and bacillopeptidase F, in the extracellular fermentation fraction of SW5 cultures, indicating the complexity and diversity of catalytic types among these extracellular proteases (20). However, little is known about the dynamics of extracellular protease activity and the regulation mechanism controlling these proteases in *B. velezensis*. In this study, we combined physiological and biochemical analyses with transcriptomics profiling of strain SW5 to investigate the dynamics of cell growth, extracellular protease activity, and the regulatory mechanisms controlling extracellular protease production. Our findings identified key regulatory factors influencing extracellular protease activity, providing valuable knowledge to enable industrial application of *B. velezensis*.

## RESULTS AND DISCUSSION

### Dynamics of cell density and extracellular protease activity in strain SW5

**Fig 1:**
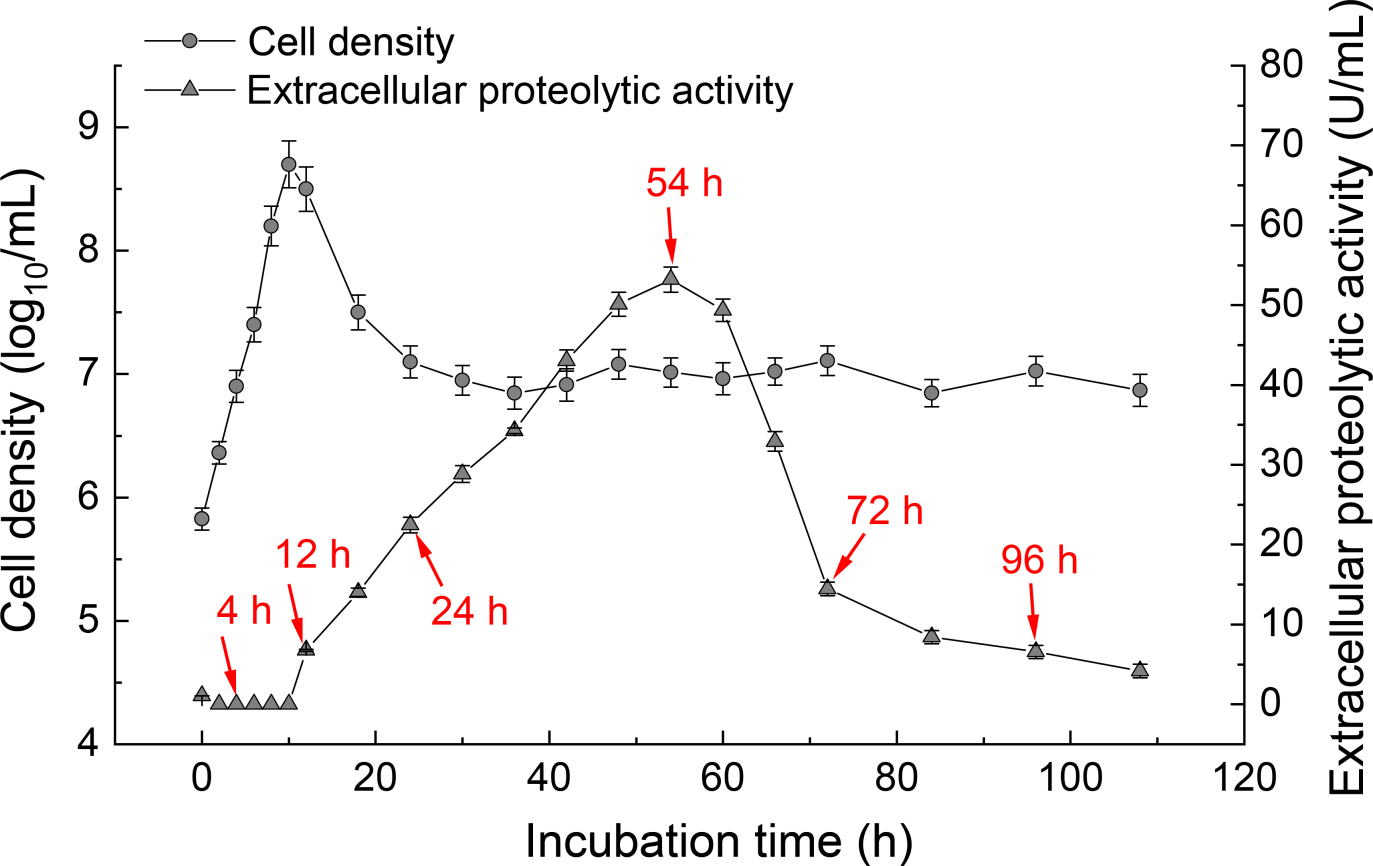
Cell density and extracellular proteolytic activity changes in strain SW5 during incubation. Cell densities (left *Y*-axis, solid circles) were measured by plate counting. The extracellular protease activity (right *Y*-axis, solid triangles) was measured by hydrolyzing casein under standard assay conditions. Values are expressed as the mean and standard deviation from three biological replicates. Red arrows indicate six time points (4, 12, 24, 54, 72, and 96 h) that were selected for transcriptomic analysis.

To investigate the growth dynamics of strain SW5, the live cell density was determined at various time points during growth. The results showed that the live cell density increased exponentially at the beginning of incubation and peaked at 10 h, followed by a decline, ultimately stabilizing after 24 h (Fig. 1). At the end of the incubation period, the density of the cell culture had increased by an order of magnitude compared to the initial cell count (Fig. 1). Based on these observations, the culture of strain SW5 could be divided into two phases: an exponential growth phase (0–10 h) and a stationary phase (10–110 h), with the transition occurring at 10 h of growth. This transition point marked the shift from exponential growth to stationary phase (21). Additionally, the extracellular protease activity was measured during the growth of strain SW5 (Fig. 1). During the exponential growth phase, the extracellular protease activity was almost undetectable (Fig. 1). When the cells reached peak density at 12 h around the transition point, the extracellular protease activity began to increase, eventually reaching its maximum at 54 h (Fig. 1). These findings are consistent with previous studies reporting that extracellular protease production and secretion are enhanced once the cell population enters the stationary phase.

### Overview of strain SW5 transcriptomes

To further investigate the regulatory mechanisms that control extracellular protease activity in strain SW5, the transcriptome was sequenced to profile the global expression pattern of SW5 genes. Based on the observed cell densities and extracellular protease activity, the culture was sampled in three biological replicates at six time points during incubation (i.e., 4, 12, 24, 54, 72, and 96 h, Fig. 1). The extracted RNA was sequenced, and the resulting data are summarized in Table S1. A total of 98.09%–99.54% of clean reads were mapped to the SW5 genome (Table S1), generating 3,784–4,114 expressed genes for each time point sample (Fig. S1). Most of the detected expressed genes (3,459 out of 4,335, 80%) were shared among all samples (Fig. S1). Notably, the 54-h sample (when extracellular protease activity peaked) had the most unique set of expressed genes (69 unique genes, Fig. S1). The principal component analysis (PCA) results showed that the transcriptomes clustered according to the sampling time points, except for the 72- and 96-h samples, which grouped together (Fig. S2). The notable variation in the transcriptomes across these time points during incubation suggested substantial changes in the global expression patterns in strain SW5 during growth.

**Fig 2:**
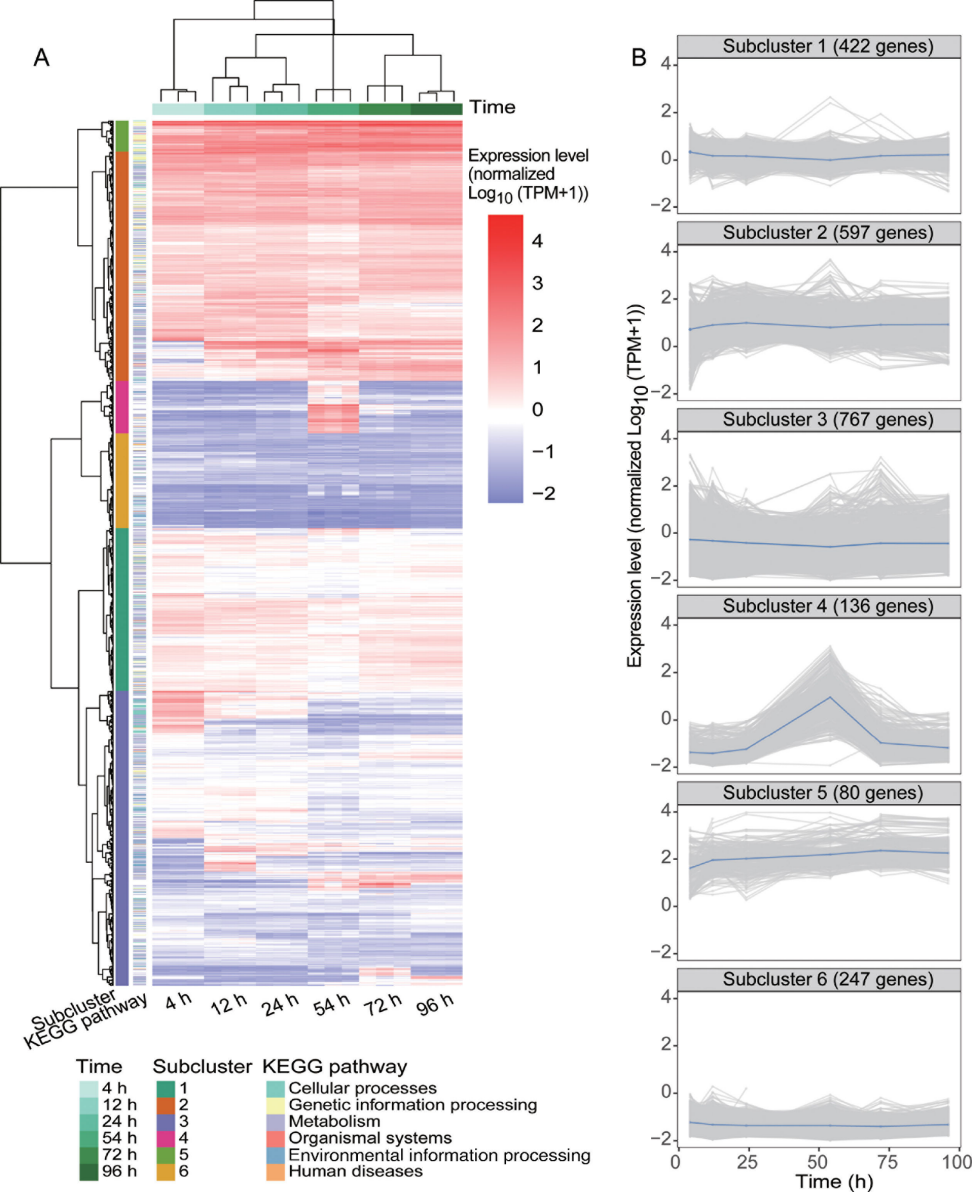
Time-course analysis of global gene expression in strain SW5 during cell growth. (**A**) Clustering of expressed genes across six time points based on their expression patterns. The gene expression levels are expressed as transcripts per million and normalized to log10 (TPM + 1), with global standardization performed using scale normalization in R. Genes with high expression levels (normalized log10 [TPM + 1] >0) are in red, while genes with low expression levels (normalized log10 [TPM + 1] < 0) are in purple. (**B**) Expression pattern of genes in subclusters 1–6.

The heatmap of the global gene expression levels revealed six subclusters of expressed genes across the sampled time points (Fig. 2), each with a unique gene expression profile. The genes in subcluster 1 (*n* = 422) maintained relatively stable average expression levels (normalized log10 [transcripts per million, TPM + 1] values near zero) throughout the growth of strain SW5. Subclusters 3 (*n* = 767) and 6 (*n* = 247) also showed relatively stable expression levels throughout growth but at lower levels (normalized log10 [TPM + 1] values < 0) compared to other subclusters. The genes in subclusters 2 (*n* = 597) and 5 (*n* = 80) had relatively stable expression levels throughout growth, with high expression levels (normalized log10 [TPM + 1] values > 0). Notably, the gene expression levels in subcluster 4 (*n* = 136) increased slightly at 12 h, peaked at 54 h, and then declined, closely mirroring the extracellular protease activity pattern. The genes belonging to nine KEGG pathways were enriched in subcluster 4 (Fig. S3, *P* < 0.05) and were primarily associated with carbohydrate metabolism, oxidative phosphorylation, amino acid degradation, and cell membrane synthesis. This indicates that the genes in subcluster 4 may participate in energy production and supply essential intermediates for extracellular protease synthesis and secretion in strain SW5. Another possibility is that the metabolic genes in subcluster 4 may also participate in nutrient scavenging as the medium became lean, and extracellular protease activity was part of this nutrient scavenging process. Overall, the expression patterns suggest that genes in subcluster 4 potentially play an important role in the production and regulation of extracellular proteases in strain SW5.

### Correlation of transcriptomes with extracellular protease activity in strain SW5

**Fig 3:**
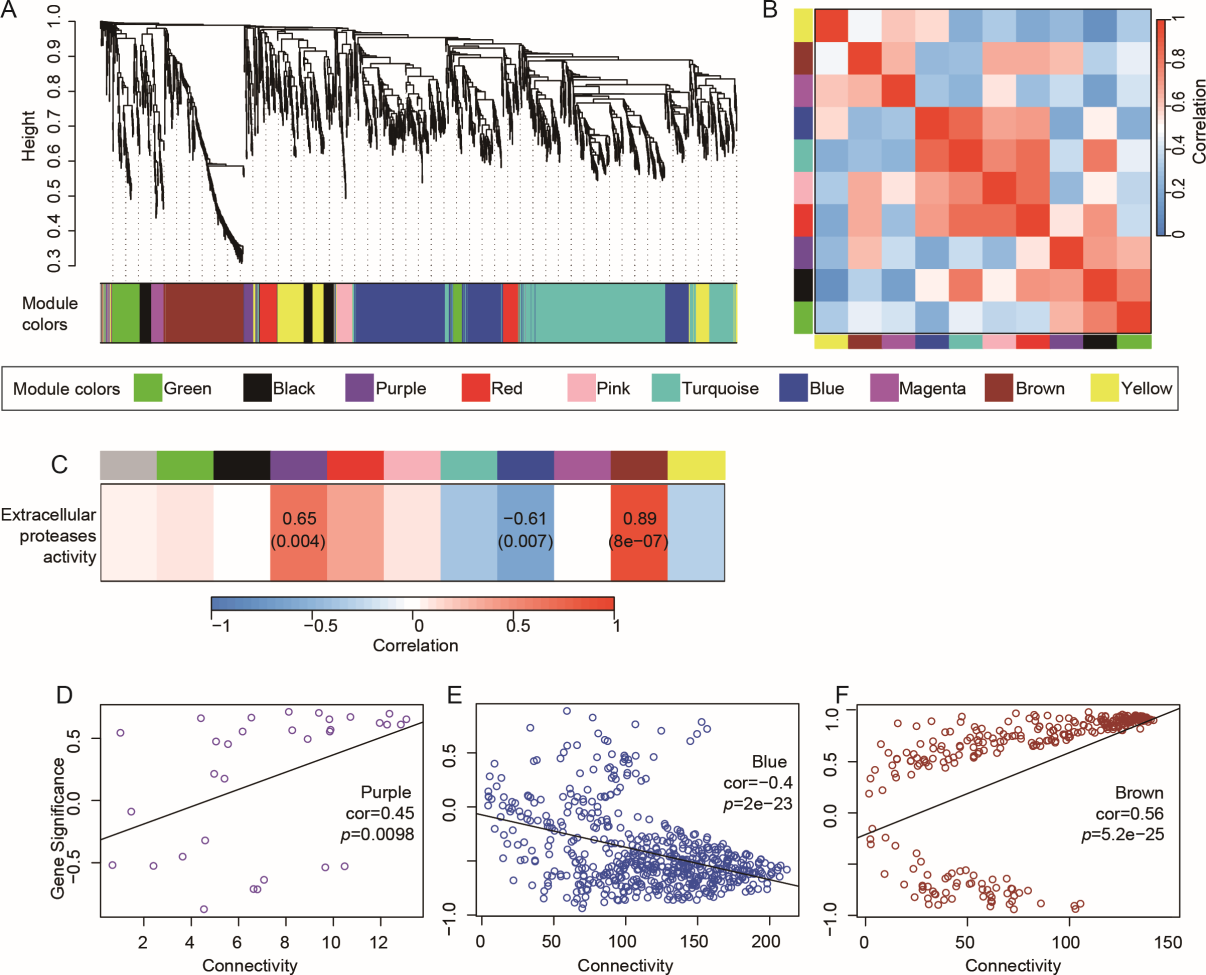
Weighted gene co-expression network analysis plots showing the correlations between gene modules and phenotypes. (**A**) Clustering of 10 gene modules identified by WGCNA. (**B**) Heatmap of correlations among WGCNA modules. (**C**) Correlations between WGCNA modules and phenotypes (extracellular protease activity). The correlation values and *P*-values are indicated for the significant correlations (*P* < 0.05). (**D–F**) Correlations between genes in purple, blue, and brown modules and extracellular protease activity.

**Fig 4:**
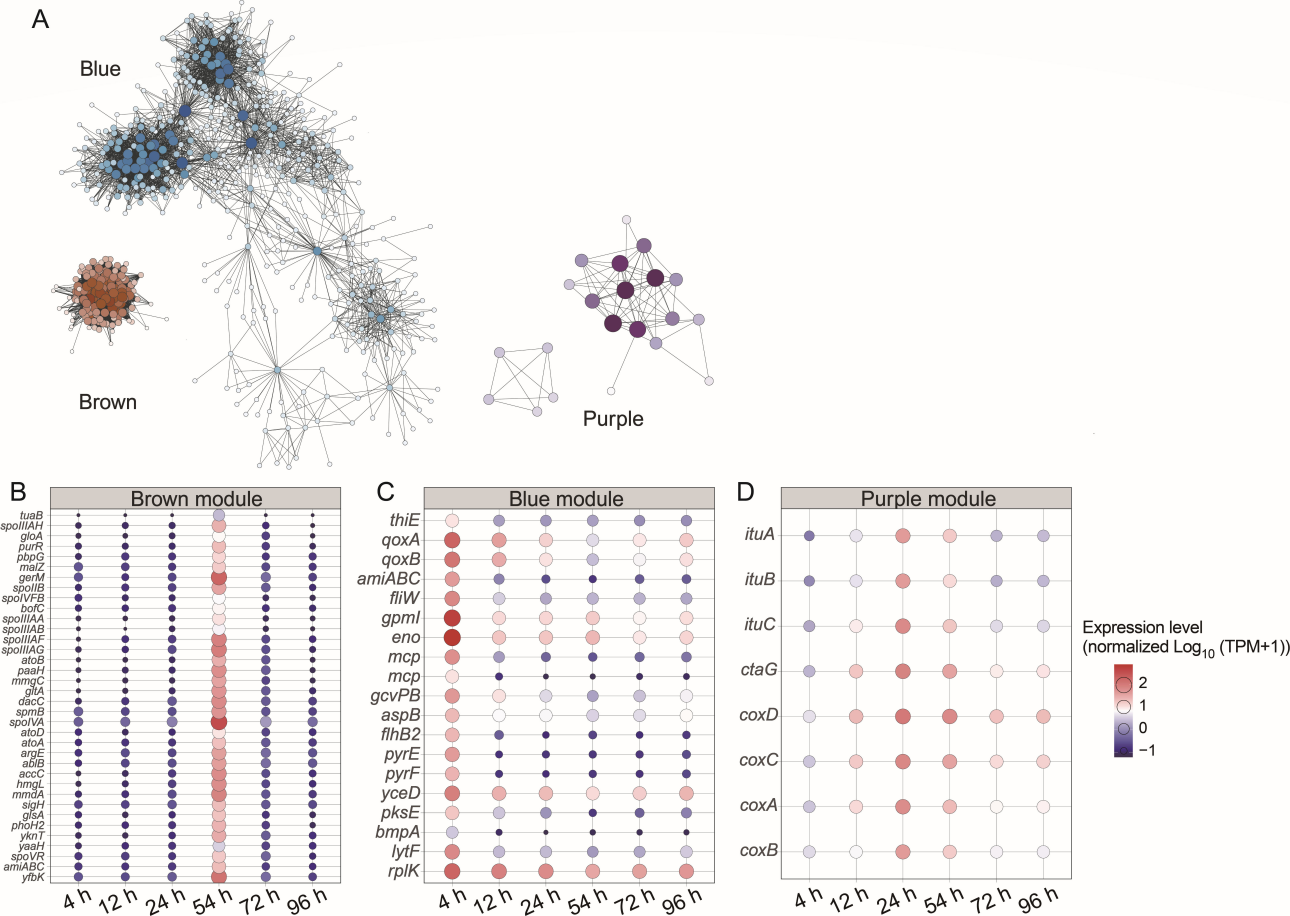
Hub genes in each module identified by weighted gene co-expression network analysis. (**A**) Correlation network of major genes in the green, brown, blue, and purple modules of WGCNA. The more links there are between genes, the larger and darker the nodes are in the network. (**B–D**) Time-course expression patterns of hub genes in the three modules. The gene expression levels are expressed in transcripts per million and normalized to log10 (TPM + 1) and global scaling. *tuaB*, teichuronic acid exporter; *spoIIIAH*, stage III sporulation protein AH; *gloA*, lactoylglutathione lyase; *purr*, purine operon repressor; *pbpG*, penicillin-binding protein 2D; *malZ*, alpha-glucosidase; *germ*, germination protein M; *spoIIB*, stage II sporulation protein B; *spoIVFB*, stage IV sporulation protein FB; *bofC*, forespore regulator of the sigma-K checkpoint; *spoIIIAA*, stage III sporulation protein AA; *spoIIIAB*, stage III sporulation protein AB; *spoIIIAF*, stage III sporulation protein AF; *spoIIIAG*, stage III sporulation protein AG; *atoB*, acetyl-CoA C-acetyltransferase; *paaH*, 3-hydroxybutyryl-CoA dehydrogenase; *mmgC*, acyl-CoA dehydrogenase; *gltA*, citrate synthase; *dacC*, serine-type D-Ala-D-Ala carboxypeptidase; *spmB*, spore maturation protein B; *spoIVA*, stage IV sporulation protein; *atoD*, acetate CoA/acetoacetate CoA-transferase alpha subunit; *atoA*, acetate CoA/acetoacetate CoA-transferase beta subunit; *argE*, acetylornithine deacetylase; *ablB*, beta-lysine N6-acetyltransferase; *accC*, acetyl-CoA carboxylase, biotin carboxylase subunit; *hmgL*, hydroxymethylglutaryl-CoA lyase; *mmdA*, methylmalonyl-CoA decarboxylase subunit alpha; *sigH*, RNA polymerase sporulation-specific sigma factor; *glsA*, glutaminase; *phoH2*, PhoH-like ATPase; *yknT*, sigma-E controlled sporulation protein; *yaaH*, spore germination protein; *spoVR*, stage V sporulation protein R; *amiABC*, N-acetylmuramoyl-L-alanine amidase; *yfbK*, Ca-activated chloride channel homolog; *thiE*, thiamine-phosphate pyrophosphorylase; *qoxA*, cytochrome aa3-600 menaquinol oxidase subunit II; *qoxB*, cytochrome aa3-600 menaquinol oxidase subunit I; *amiABC*, N-acetylmuramoyl-L-alanine amidase; *fliW*, flagellar assembly factor FliW; *mcp*, methyl-accepting chemotaxis protein; *gcvPB*, glycine dehydrogenase subunit 2; *aspB*, aspartate aminotransferase; *flhB2*, flagellar biosynthesis protein; *pyrE*, orotate phosphoribosyltransferase; *pyrF*, orotidine-5′-phosphate decarboxylase; *yceD*, DUF177 domain-containing protein; *pksE*, trans-AT polyketide synthase, acyltransferase, and oxidoreductase domains; *bmpA*, basic membrane protein A and related proteins; *lytF*, peptidoglycan DL-endopeptidase LytF; *rplK*, large subunit ribosomal protein L11; *ituA*, iturin family lipopeptide synthetase A; *ituB*, iturin family lipopeptide synthetase B; *ituC*, iturin family lipopeptide synthetase C; *ctaG*, putative membrane protein; *coxD*, cytochrome c oxidase subunit IV; *coxC*, cytochrome c oxidase subunit III; *coxA*, cytochrome c oxidase subunit I; and *coxB*, cytochrome c oxidase subunit II.

To elucidate correlations between the gene expression levels and regulatory networks in strain SW5, weighted gene co-expression network analysis (WGCNA) was performed to create a co-expression network, which organized the genes into 10 distinct modules (Fig. 3). Notably, an additional module-phenotype correlation analysis revealed three core modules correlated with the extracellular protease activity of strain SW5 (Fig. 3C). Significant correlations were found between the purple, blue, and brown modules and the extracellular protease activity (purple Cor = 0.65, *P* < 0.01; blue Cor = −0.61, *P* < 0.01; and brown Cor = 0.89, *P* < 0.01, Fig. 3C). Similarly, correlations between all genes within these modules and the extracellular protease activity were also significant (purple Cor = 0.45, *P* < 0.01; blue Cor = −0.40, *P* < 0.01; and brown Cor = 0.56, *P* < 0.01, Fig. 3D through F). The genes in the blue module displayed high expression levels during the exponential growth phase (Fig. 4C) and were enriched in ribosome synthesis, aminoacyl-tRNA biosynthesis, and carbon metabolism pathways (Fig. S4A). These pathways correspond to the high metabolic demands during the exponential growth phase. This module contains seven extracellular protease genes, three of which carry signal peptides: *bpr*, *aprE*, and *epr* (encoding minor extracellular protease). Among them, AprE is a key industrial protease, which could be widely used in various industrial applications (22, 23). *Bacillus* is the primary production strain for AprE because it is generally recognized to be safe and harbor favorable growth characteristics (4). Additionally, AprE can also be produced in engineered *Pichia* and molds, further enhancing its biotechnological versatility (22, 24). Genes that were potentially regulating *aprE* expression, such as *comA*, were also identified in the blue module and displayed similar expression patterns, thus further supporting their close correlation in expression regulation. The genes in the brown module shared significant overlap with those in subcluster 4 (Fig. 2) and were primarily involved in KEGG pathways related to peptidoglycan biosynthesis, butanoate metabolism, and lysine degradation (Fig. S4B). The genes in the purple module were primarily involved in the oxidative phosphorylation and nonribosomal peptide synthesis pathways, along with only one signal peptide-carrying endopeptidase gene, *cwlS* (encoding peptidoglycan DL-endopeptidase, Fig. S4C). Similarly, the hub genes in the purple module were mainly involved in nonribosomal peptide structures and oxidative phosphorylation (Fig. 4D). Oxidative phosphorylation, which is a key step that follows glycolysis and the TCA cycle, efficiently generates ATP and directly contributes to the energy needs of strain SW5. Overall, the correlation between expression of these genes in the blue, brown, and purple modules and extracellular protease activity is suggestive of their potential cooperative roles in producing and secreting extracellular proteases in strain SW5.

### Potential regulation mechanism of extracellular protease activity

**Fig 5:**
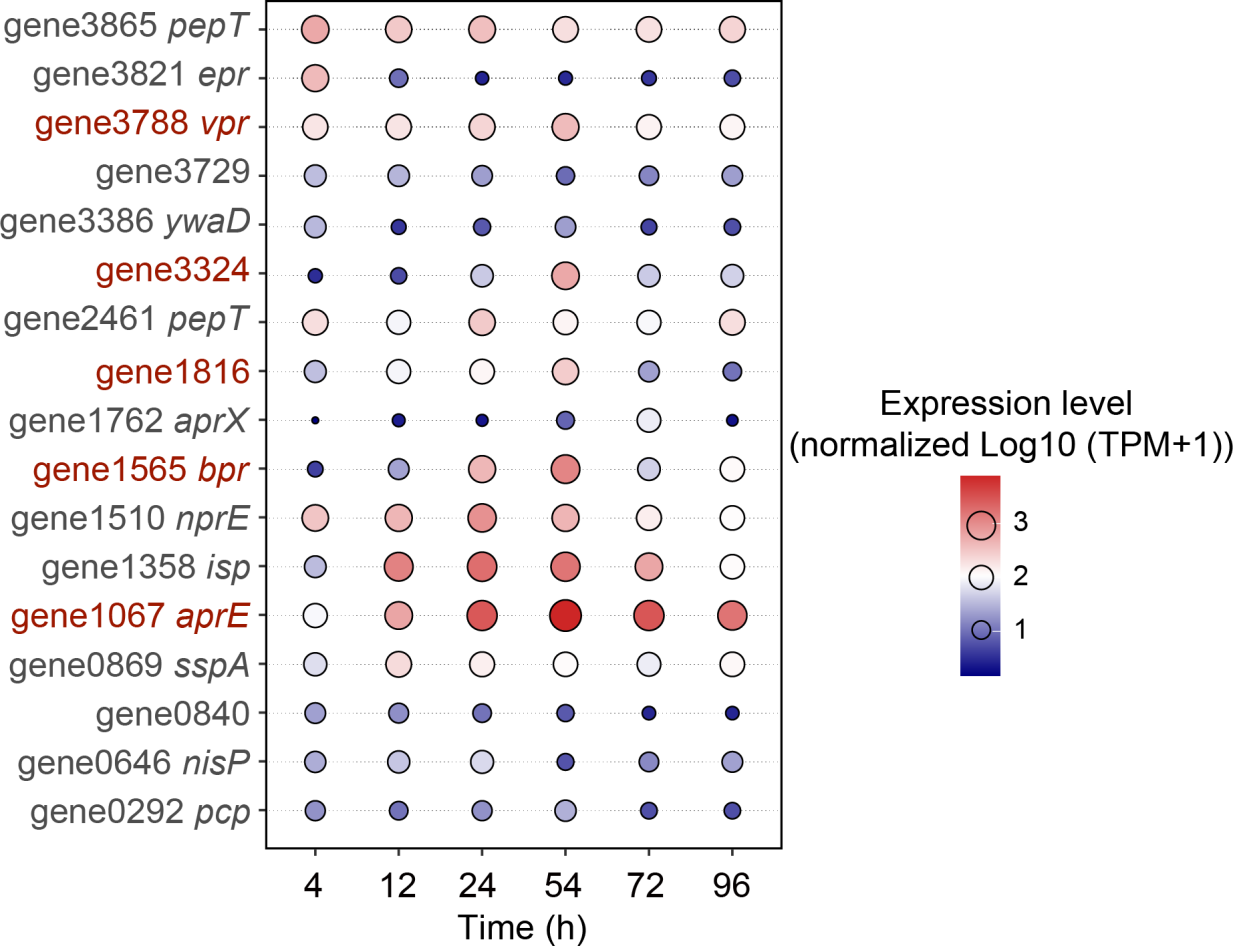
Expression pattern of 17 extracellular proteases in strain SW5. The gene expression levels are expressed in transcripts per million and normalized to log10 (TPM + 1) and global scaling. Eight extracellular proteases that had high expression levels at 54 h are colored in red. *pepT*, tripeptide aminopeptidase; *epr*, minor extracellular protease; *vpr*, minor extracellular protease; gene3729, zinc-dependent protease; *ywaD*, aminopeptidase YwaD; gene3324, zinc-dependent metalloprotease; *pepT*, tripeptidase T; gene1816, peptidase G2; *aprX*, serine protease; *bpr*, bacillopeptidase F; *nprE*, metallopeptidase; *isp*, major intracellular serine protease; *aprE*, serine protease; *sspA*, serine protease; gene0840, peptidase G2; *nisP*, serine peptidase; and *pcp*, pyroglutamyl peptidase.

To determine the key extracellular proteases contributing to the total extracellular protease activity of SW5, we analyzed the expression patterns of 17 extracellular protease-encoding genes (Table S2) throughout the growth of strain SW5. The results showed that eight extracellular proteases had relatively high expression levels at 54 h (Fig. 5). Among them, *aprE* (subtilisin E) had the highest expression level and exhibited an expression pattern closely mirroring the extracellular protease activity pattern of strain SW5. Collectively, the result indicates that AprE is a key extracellular protease contributing to the total extracellular protease activity of strain SW5.

**Fig 6:**
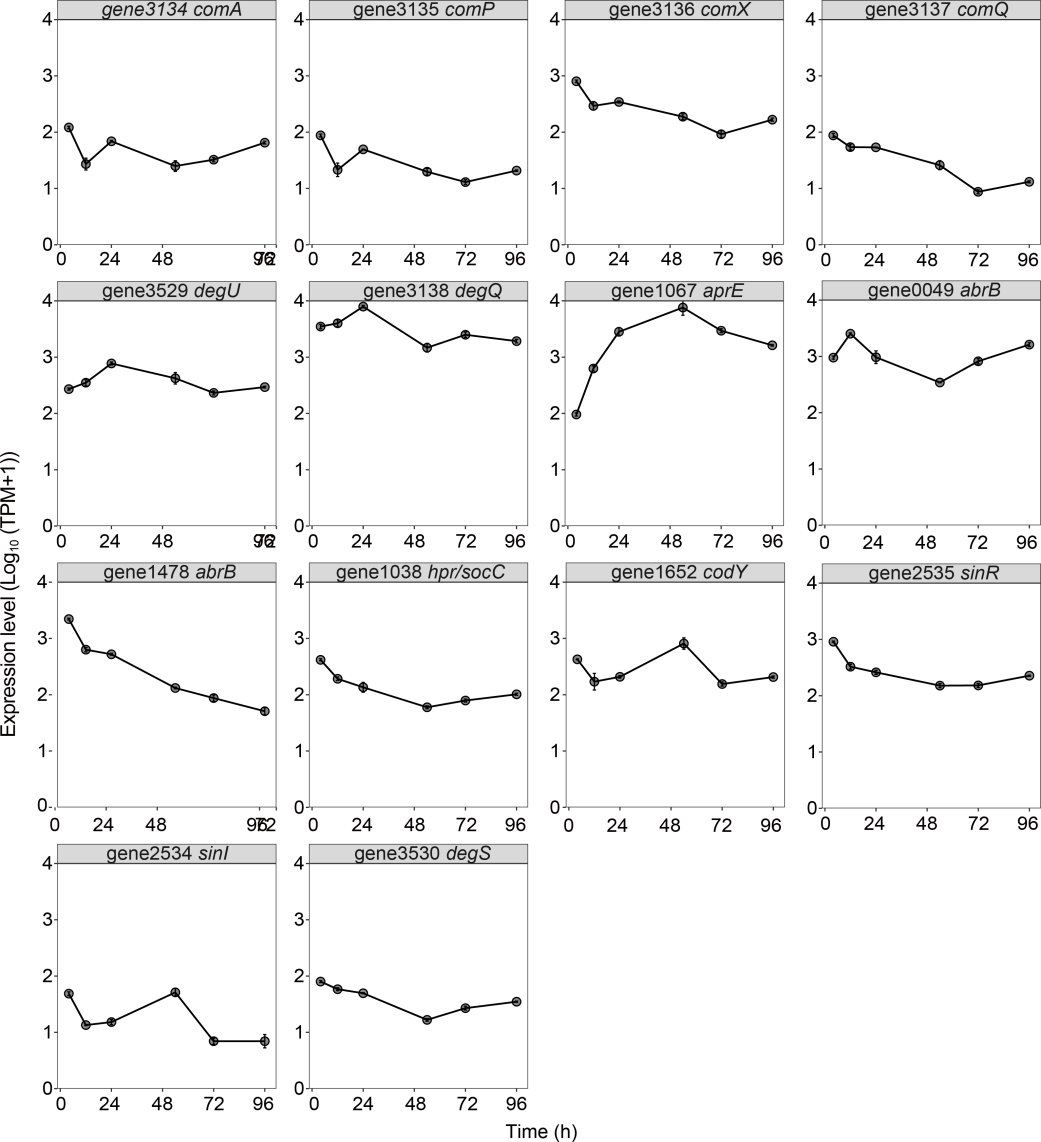
Time-course expression profiles of key genes in the extracellular protease regulation network, including the *comQXPA* operon, two-component system, *degQ*, negative regulators, and key extracellular proteases (e.g., *aprE*). Expression levels are expressed in transcripts per million, normalized as log10 (TPM + 1). *comA*, response regulator transcription factor; *comP*, sensor histidine kinase; *comX*, competence pheromone; *comQ*, polyprenyl synthetase family protein; *degU*, two-component system response regulator; *degQ*, degradation enzyme regulation protein; *aprE*, serine protease; *abrB*, AbrB family transcriptional regulator; *hpr/socC,* protease production regulatory protein; *codY*, transcriptional pleiotropic repressor; *sinR*, XRE family transcriptional regulator; *sinI*, antagonist of SinR; and *degS*, sensor histidine kinase.

**Fig 7:**
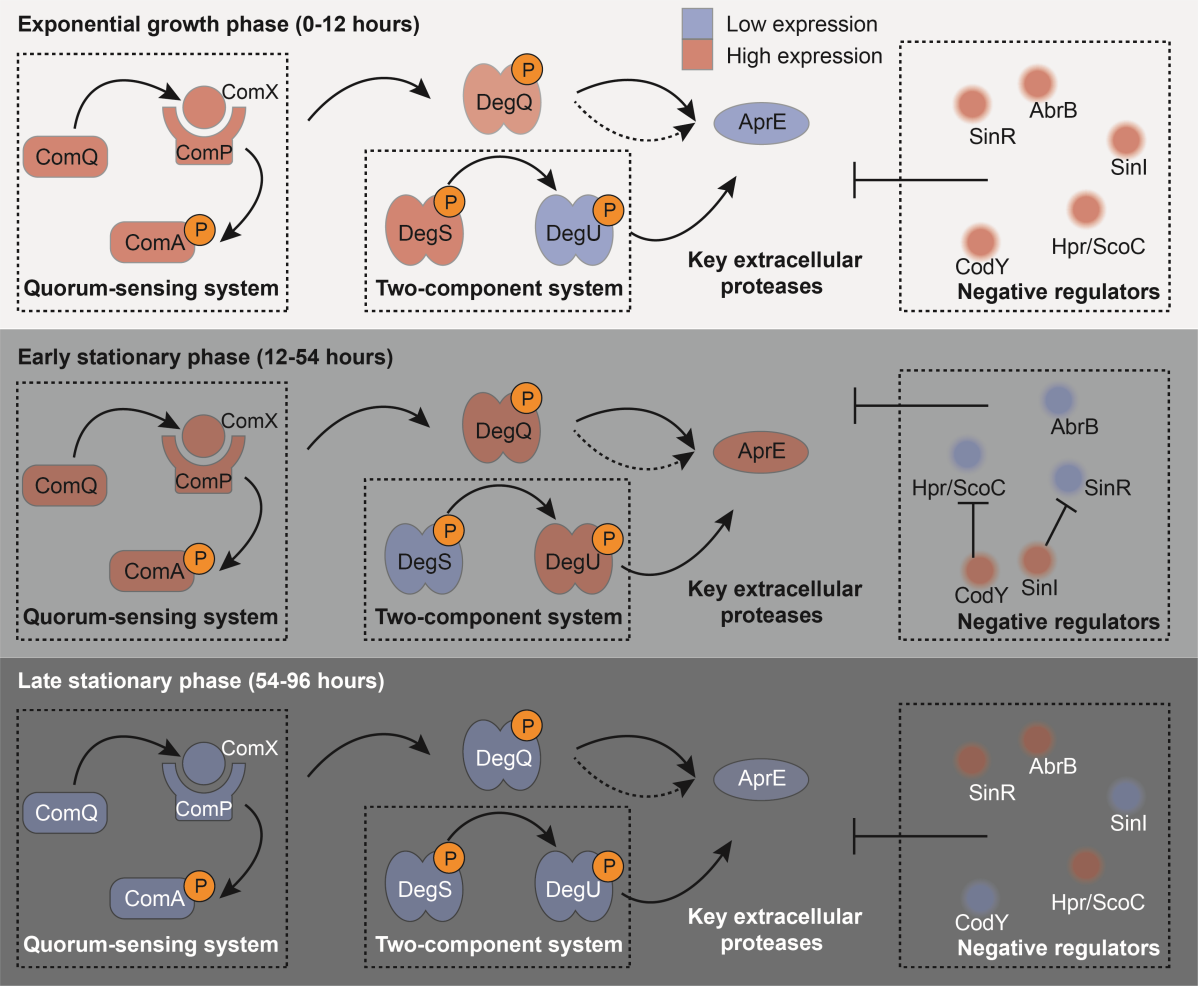
A proposed model describing the regulation of key extracellular proteases by the *comQXPA* operon, two-component system, *degQ*, and negative regulators in strain SW5 at different growth phases. During the exponential growth phase (4–12 h), the low concentration of phosphorylated DegU and the high expression of negative regulators (*abrB*, *hpr*/*scoC, codY, sinR,* and *sinI*) inhibit the expression of key extracellular proteases (e.g., AprE). During the early stationary phase (12–54 h), the high expression of the *comQXPA* operon, *degQ*, and *degU* increases the concentration of phosphorylated DegQ and DegU, enhancing the expression of *aprE*. Meanwhile, the inhibition of *hpr*/*scoC* and *sinR* by highly expressed *codY* and *sinI*, as well as the low expression of *abrB*, further enhances the expression of *aprE*. During the late stationary phase (54–96 h), the decreased concentration of phosphorylated DegU and DegQ, along with the high expression of negative regulators (*abrB*, *hpr*/*scoC,* and *sinR),* inhibits the expression of *aprE*. The genes with high or low expression levels are colored in red or blue, respectively. Solid black arrows indicate direct regulation, and dashed black arrows indicate indirect regulation. The T-shaped line represents inhibition. *comA*, response regulator transcription factor; *comP*, sensor histidine kinase; *comX*, competence pheromone; *comQ*, polyprenyl synthetase family protein; *degU*, two-component system response regulator; *degQ*, degradation enzyme regulation protein; *degS*, sensor histidine kinase; *aprE*, serine protease; *abrB*, AbrB family transcriptional regulator; *hpr/socC,* protease production regulatory protein; *codY*, transcriptional pleiotropic repressor; *sinR*, XRE family transcriptional regulator; and *sinI*, antagonist of SinR.

To explore the regulation mechanisms that control extracellular proteases, we further investigated the expression patterns of key regulator genes, including the *comQXPA* operon, *degU*, *degQ*, and several negative regulators (Fig. 6). The genes in the *comQXPA* operon (i.e., *comQ*, *comX*, *comP*, and *comA*) exhibited similar expression patterns (Fig. 6), which were consistent with the activation mode of the *comQXPA* operon reported in *B. subtilis* (12, 15, 25). Based on the temporal expression pattern of these regulators and key extracellular proteases (e.g., *aprE*, Fig. 6), we propose a model that describes the potential regulation mechanisms of extracellular protease production by the *comQXPA* operon, *degU*, *degQ*, and negative regulators in strain SW5 at different phases of growth (Fig. 7). During the exponential growth phase (4–12 h), the low expression levels of *degU* likely lead to the low concentration of phosphorylated DegU, thereby inhibiting the expression of extracellular proteases. Meanwhile, the high expression levels of the negative regulators gene1478_*abrB,* gene0049_*abrB*, *hpr*/*scoC, codY, sinR,* and *sinI* during the exponential growth phase also likely contribute to the inhibition of extracellular proteases. Moreover, during the early stationary phase (12–54 h), the high expression levels of the *comQXPA* operon, *degQ,* and *degU* likely lead to the high concentration of activated DegQ and phosphorylated DegU, thereby enhancing the expression of *aprE*. In addition, the gradual drop in the expression levels of the negative regulators, gene1478_*abrB,* gene0049_*abrB*, *hpr*/*scoC,* and *sinR,* during the early stationary phase likely relieves the inhibition of extracellular proteases. The negative regulators *codY* and *sinI* were highly expressed during the early stationary phase, and their expression peaked at 54 h. Previous studies have shown that *codY* and *sinI* inhibit *hpr*/*scoC* and *sinR*, respectively, when the *codY* and *sinI* expression levels are at their highest, which, in turn, enhances the expression of extracellular proteases (26, 27). Collectively, the above-combined effects of increased DegQ and DegU phosphorylation and the suppression of negative regulators during the early stationary phase likely lead to the dramatic increase in the extracellular protease activity, which peaks at 54 h. During the late stationary phase (54–96 h), the lower expression levels of the *comQXPA* operon likely lead to the lower concentrations of phosphorylated DegU and activated DegQ, thus inhibiting the expression of *aprE*. Meanwhile, the high expression levels of the negative regulators gene0049_*abrB*, *hpr*/*scoC,* and *sinR* during the exponential growth phase also likely contribute to the inhibition of the extracellular proteases during this period. Taken together, these results show that extracellular proteases in *B. velezensis* are likely tightly regulated by a complex network involving the DegS-DegU two-component system, ComQXPA quorum-sensing system, and negative regulators during the different phases of growth (Fig. 7). In general, the regulatory model of extracellular proteases in strain SW5 is similar to that of *B. subtilis*, as they share most of the regulatory elements, such as DegQ, DegU, ComQXPA quorum-sensing system, and negative regulators (e.g., *abrB*, *hpr/scoC*, and *codY*). However, our study revealed the growth stage-specific regulation of extracellular proteases in strain SW5, which has not been reported in *B. subtilis*. In addition, Spo0A serves as a critical global regulator for extracellular proteases in *B. subtilis* (11), while our transcriptomic data showed that Spo0A may not play key roles in the regulation of extracellular proteases in strain SW5. However, it is worth noting that this proposed regulatory model in strain SW5 is generated just based on a correlation in gene expression, and future experimental studies are needed to verify this proposed model.

### Conclusions

In this study, we systematically investigated the dynamics of cell density and extracellular protease activity, as well as potential regulatory mechanisms of extracellular protease production in strain SW5 during growth. Physiological assays showed that SW5’s cell density and extracellular protease activity displayed opposite trends. Transcriptomic analysis revealed distinct expression patterns among various extracellular protease genes and their regulators at different stages of cell growth, highlighting the complexity of the regulation network that controls extracellular protease activity in strain SW5. In particular, several extracellular proteases (e.g., AprE) significantly contributed to the extracellular protease activity of strain SW5, which was collectively regulated by the DegS-DegU two-component system, the ComQXPA quorum-sensing system, DegQ, and negative regulators. Taken together, these findings provide valuable knowledge on the regulatory mechanisms governing extracellular protease activity in *B. velezensis*.

## MATERIALS AND METHODS

### Strain culturing and measurement of cell density and extracellular protease activity

*B. velezensis* SW5 was inoculated in MC medium [yeast extract, 2.5 g/L; casein, 2.0 g/L; glucose, 5.0 g/L; (NH_4_)_2_SO_4_, 2.0 g/L; K_2_HPO_4_, 14.0 g/L; KH_2_PO_4_, 6.0 g/L; MgSO_4_, 0.2 g/L; soluble starch, 22 g/L; CaCl_2_, 3 mmol/L; and a pH of 6.0] and incubated at 35°C while shaking at 200 rpm. Sampling was conducted every 2 h during the early growth phase and every 6 h during the late growth phase to monitor the cell density and extracellular protease activity. Cell growth was assessed using the plate count method to determine the cell density. The extracellular protease activity was measured as described previously (28). In brief, the enzymatic reaction was conducted at 40°C for 10 minutes, with casein as the substrate. One unit of enzyme was defined as the amount of enzyme required to hydrolyze casein to release 1 µg of tyrosine per minute under the above-mentioned conditions.

### RNA extraction, library construction, and sequencing

For transcriptome profiling, SW5 cells from different time point samples were collected by centrifugation at 8,000 rpm for 15 minutes at 4°C, and the cell pellets were immediately flash-frozen in liquid nitrogen and stored at −80°C until RNA extraction. Total RNA was extracted using TRIzol reagent according to the manufacturer’s instructions (Invitrogen Life Technologies, USA). Following quality control, rRNA was removed from the total RNA using the RiboCop rRNA Depletion Kit for Mixed Bacterial Samples (Lexogen, USA). The resulting mRNA was randomly sheared into fragments with lengths of approximately 200 bp. Double-stranded cDNA was synthesized from mRNA templates using reverse transcriptase and random hexamer primers (Illumina Inc., USA). During the synthesis of the second strand, dUTP was used instead of dTTP. The end-repair, phosphorylation, and “A” overhang addition were performed according to Illumina’s library construction protocol. The RNA-seq libraries were prepared using the Illumina Stranded mRNA Prep Ligation (San Diego, USA), and sequencing was done on the Illumina Novaseq 6000 (Illumina Inc., USA).

### RNA-seq data analysis

Raw reads were filtered using Fastp version 0.19.6 (length > 25 bp, *Q* > 20, and no N bases) (29). Subsequently, 10,000 random clean reads from each sample were aligned with the Rfam database using BLAST to assess the percentage of rRNA contamination. The clean reads were then mapped to the genome of strain SW5 (accession number NZ_CP074348.1) using Bowtie version 2.3.4 with default parameters (30). The genes of strain SW5 were annotated using the NCBI NR, GO, and KEGG databases (*e*-value < 1e^−5^) to obtain information on gene function and classification. Read counts for each gene were calculated using FeatureCounts (31). To determine the gene expression levels, reads per kilobase million and transcripts per million were calculated using RSEM (32, 33), and then they were log10-transformed. The heatmap and bubble were plotted by log10-transforming TPM and further globally using scale normalization in R, which ensured that the data were standardized across all time points. Gene expression patterns were examined using weighted correlation network analysis (version 1.72.5) based on log10-normalized TPM (34). A standard workflow was applied to minimize noise, and an adjacency matrix was constructed with a soft threshold of 12. Modules were identified using the dynamic tree cut algorithm, with a minimum module size of 30 and a merge threshold of 0.25 (34). Hub genes were identified based on their connectivity to module eigengenes (35). The networks were visualized in Cytoscape version 3.10.2. Differentially expressed genes (DEGs) from pair-wise comparisons among time point samples were identified using DESeq2 version 1.42.0 (*P*-value < 0.01 and |log2FC| > 1) (36). Candidate genes, including DEGs, key module genes from WGCNA, and genes clustered through temporal expression analysis, which were enriched in the GO and KEGG databases, were identified using clusterProfiler version 4.10.1 (*P*-value ≤ 0.05) (37, 38). Protease-related genes were manually selected based on genome annotation information and experimental protease activities reported in the literature. Signal peptides of proteases were predicted using the UniProtKB database annotation (https://www.uniprot.org/) and the online tool SignalP-6.0 (https://services.healthtech.dtu.dk/services/SignalP-6.0/) (39, 40). The resulting extracellular proteases investigated in this study are listed in Table S2.

Matrix data processing was performed using R packages tibble version 3.2.1, dplyr version 1.1.4, and Rmisc version 1.5.1. Line charts, PCA plots, and bubble plots were generated using the R packages RColorBrewer version 1.1.3 and ggplot2 version 3.5.1. Heatmaps were generated using the R package pheatmap version 1.0.12. Venn diagrams and upset plots were generated using TBtools version 1.120 (41).

## Data Availability

Raw transcriptome data are deposited in the NCBI SRA database under BioProject number PRJNA1195724.

## SUPPLEMENTAL MATERIAL

Supplemental material aem.01294-25-s0001.docx Figures S1 to S4; Tables S1 and S2.

ASM does not own the copyrights to Supplemental Material that may be linked to, or accessed through, an article. The authors have granted ASM a non-exclusive, world-wide license to publish the Supplemental Material files. Please contact the corresponding author directly for reuse.
