# Supplementary material for: Activity dynamics and regulation mechanism of extracellular proteases in Bacillus velezensis SW5
Source: Appl Environ Microbiol. 2025 Oct 14;91(11):e01294-25. doi: 10.1128/aem.01294-25 (PMC12628753; doi:10.1128/aem.01294-25)
Supplement: Supplemental material — Figures S1 to S4; Tables S1 and S2. [file aem.01294-25-s0001.docx]

**Supplemental material**

**The** **activity dynamics and regulation mechanism of extracellular proteases in *Bacillus velezensis* SW5**

Xuejin Feng^1†^, Yang Liu^2,3†^, Ruolin Chen^1*^, Min Jin^1*^

^1^State Key Laboratory Breeding Base of Marine Genetic Resource, Third Institute of Oceanography, Ministry of Natural Resources, 361005, Xiamen, China

^2^Institute of Agricultural Quality Standards and Testing Technology/Fujian Key Laboratory of Agro-Products Quality and Safety, Fujian Academy of Agricultural Sciences, Fuzhou 350003, PR China

^3^Fujian Key Laboratory of Special Aquatic Formula Feed, Fuzhou 350308, PR China

† These authors contributed equally to this work.

*Corresponding author (Min Jin):

E-mail address: [Jinmin@tio.org.cn](mailto:Jinmin@tio.org.cn)

*Corresponding author (Ruolin Chen):

E-mail address: Chenruolin@tio.org.cn

### Supplemental Figure


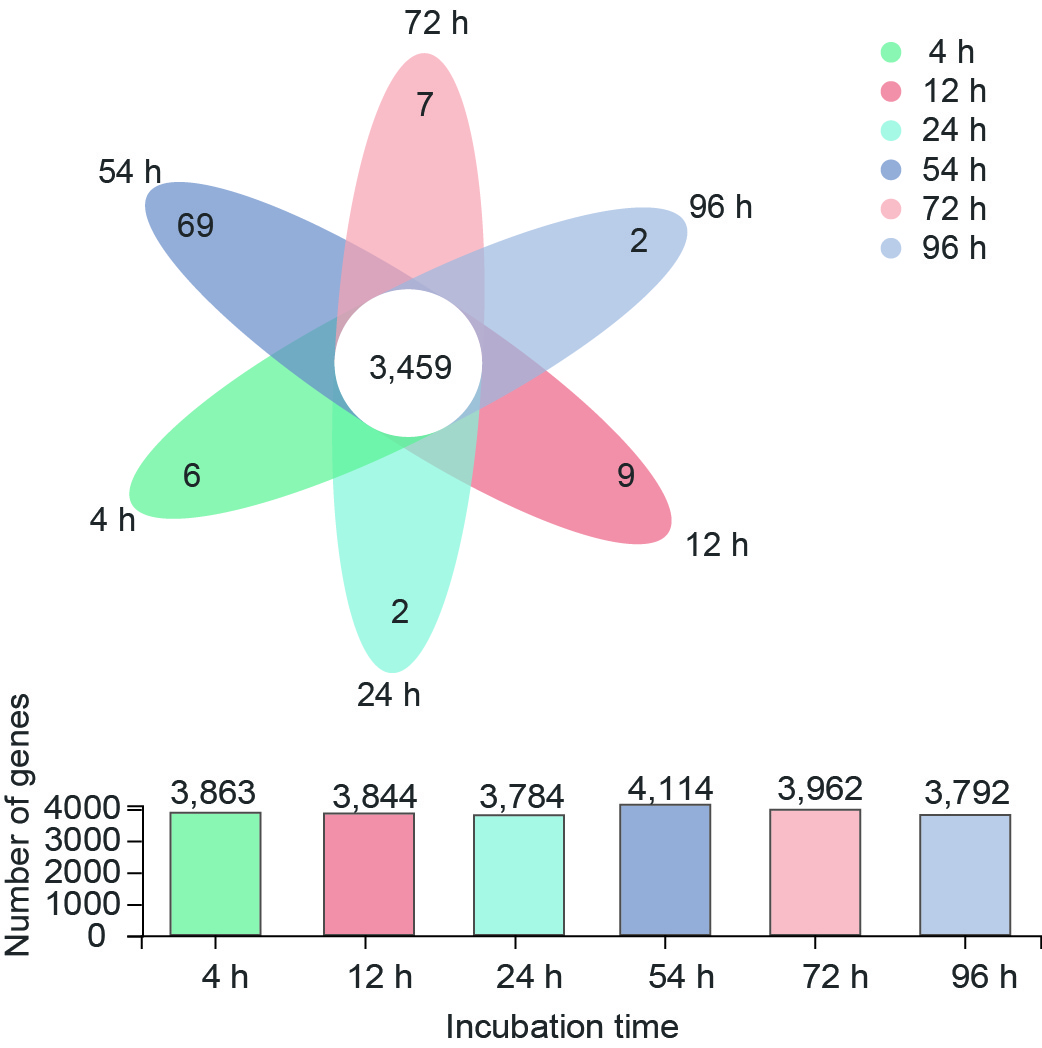


Fig. S1 Venn diagram (above) and bar chart (bottom) of expressed genes in strain SW5 across six time points. The venn diagram shows the number of unique and shared expressed genes among time point samples. The bar chart shows the number of expressed genes in each time point sample.


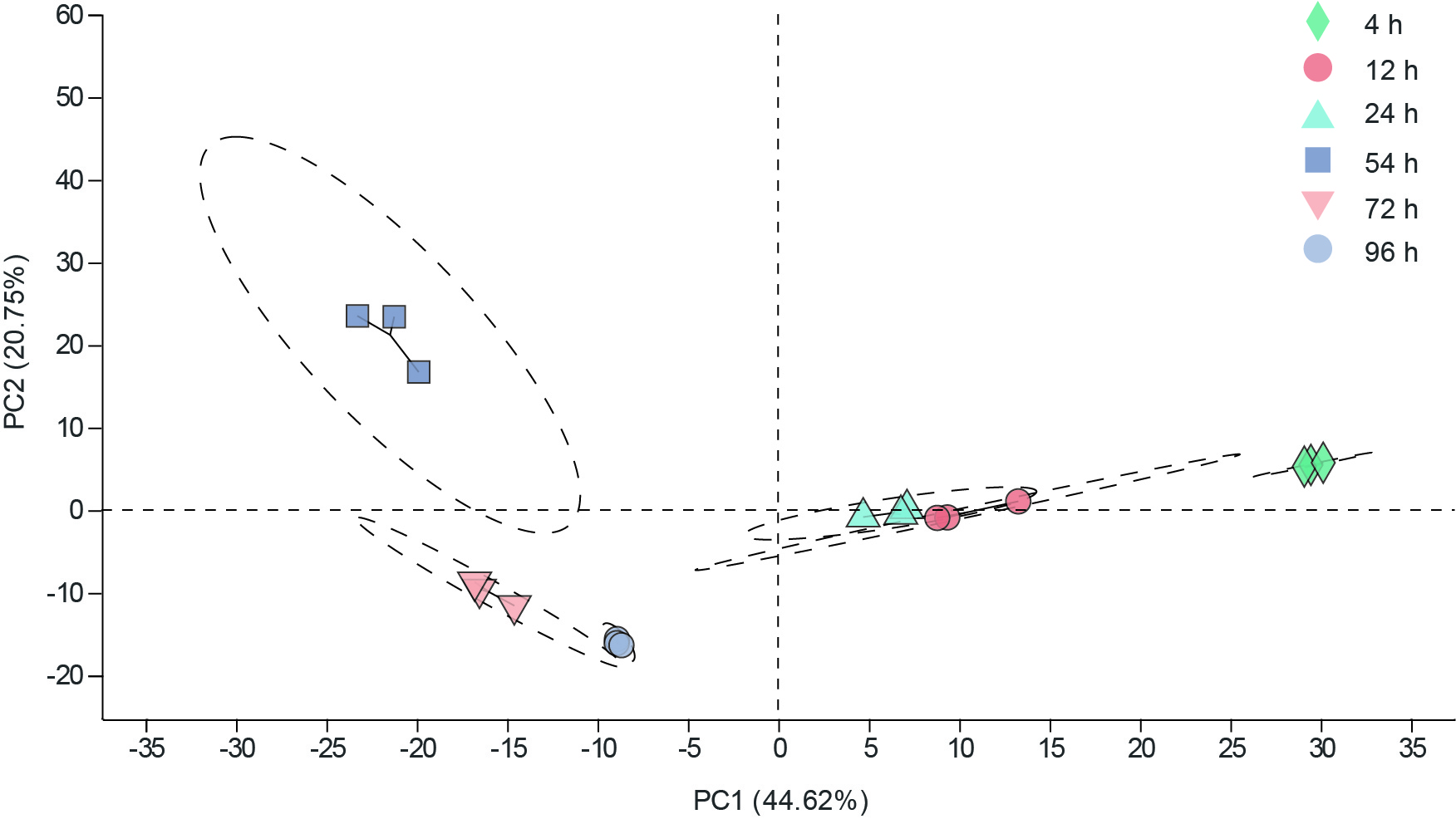


Fig. S2 Principal component analysis (PCA) of strain SW5 transcriptomes. The distances among the samples represent the level of similarity among the samples, with closer proximity indicating higher similarity. The x-axis represents the contribution of Principal Component 1 (PC1) to the differentiation of the samples, while the y-axis shows the contribution of Principal Component 2 (PC2) in distinguishing the samples.


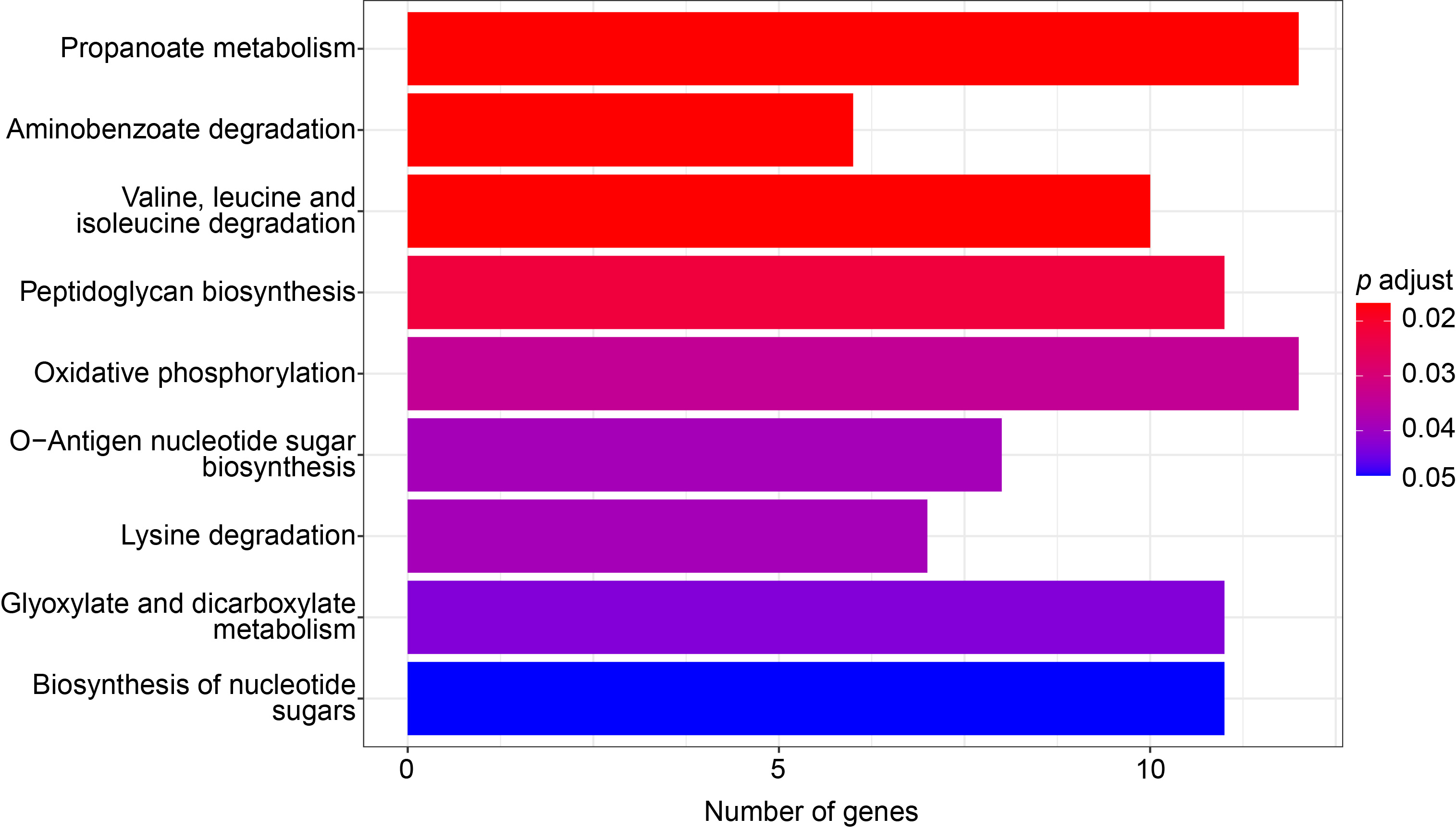


Fig. S3 KEGG enrichment results for genes in subcluster 4. The adjusted *p*-value and number of genes in each enriched pathway are indicated.


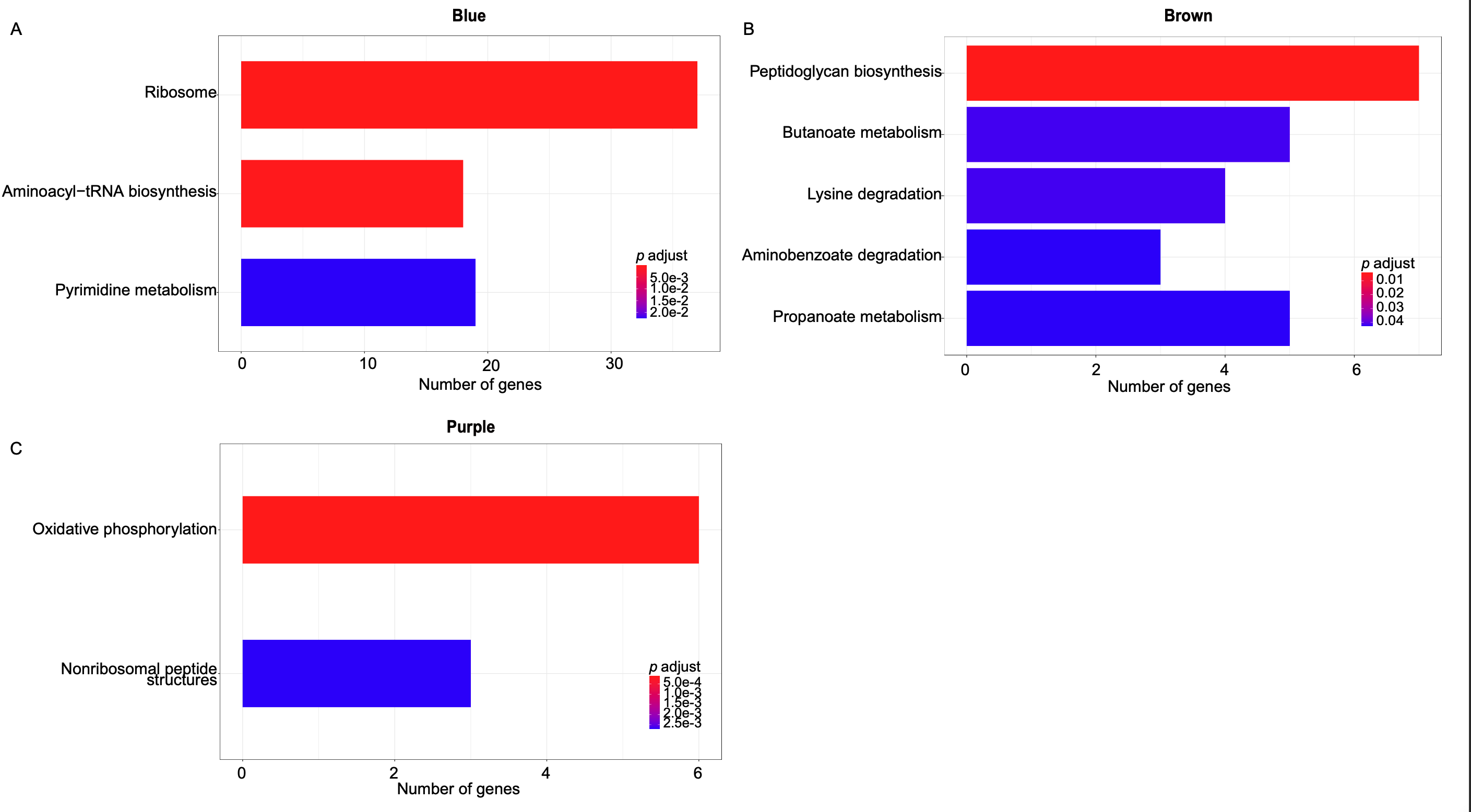


Fig. S4 KEGG pathway enrichment analysis for the genes in the blue (a), brown (b), and purple modules (c) identified in the weighted gene co-expression network.

### Supplemental Table

Table S1 Overview of transcriptome data

| Sample ID | Raw reads | Raw bases | Clean reads | Clean bases | Clean Q20 (%) | Clean Q30 (%) | GC (%) | rRNA Ratio (%) | Mapped reads | Mapped ratio (%) |
| --- | --- | --- | --- | --- | --- | --- | --- | --- | --- | --- |
| MC1_4h | 24,087,400 | 3,637,197,400 | 23,945,824 | 3,219,724,231 | 97.97 | 94.18 | 46.53 | 2.211 | 23,815,561 | 99.46 |
| MC2_4h | 22,900,750 | 3,458,013,250 | 22,764,478 | 3,070,308,658 | 97.98 | 94.18 | 46.61 | 2.303 | 22,660,571 | 99.54 |
| MC3_4h | 23,704,148 | 3,579,326,348 | 23,560,312 | 3,174,177,710 | 97.96 | 94.17 | 46.44 | 2.105 | 23,443,144 | 99.50 |
| MC1_12h | 23,791,470 | 3,592,511,970 | 23,623,494 | 3,076,686,594 | 98.14 | 94.66 | 47.7 | 5.969 | 23,461,893 | 99.32 |
| MC2_12h | 26,372,302 | 3,982,217,602 | 26,192,882 | 3,348,644,035 | 98.29 | 94.99 | 48.21 | 8.142 | 25,922,010 | 98.97 |
| MC3_12h | 26,779,074 | 4,043,640,174 | 26,570,416 | 3,383,745,602 | 98.25 | 94.93 | 48.12 | 10.472 | 26,172,400 | 98.50 |
| MC1_24h | 25,022,672 | 3,778,423,472 | 24,840,380 | 3,230,340,762 | 98.11 | 94.54 | 46.98 | 4.669 | 24,644,323 | 99.21 |
| MC2_24h | 26,061,326 | 3,935,260,226 | 25,874,896 | 3,340,015,422 | 98.21 | 94.8 | 47.1 | 5.378 | 25,725,989 | 99.42 |
| MC3_24h | 26,575,768 | 4,012,940,968 | 26,383,214 | 3,415,731,417 | 98.09 | 94.46 | 47.16 | 4.449 | 26,178,782 | 99.23 |
| MC1_54h | 26,010,200 | 3,927,540,200 | 25,830,804 | 3,380,612,252 | 98.01 | 94.33 | 47.04 | 2.657 | 25,700,651 | 99.50 |
| MC2_54h | 23,473,514 | 3,544,500,614 | 23,318,230 | 3,069,550,488 | 98 | 94.33 | 46.92 | 2.021 | 23,181,414 | 99.41 |
| MC3_54h | 23,264,348 | 3,512,916,548 | 23,103,948 | 3,019,325,295 | 98.02 | 94.39 | 47.26 | 3.299 | 22,996,294 | 99.53 |
| MC1_72h | 23,254,512 | 3,511,431,312 | 23,171,388 | 3,073,244,673 | 98.58 | 95.57 | 46.33 | 0.684 | 23,053,577 | 99.49 |
| MC2_72h | 24,348,820 | 3,676,671,820 | 24,254,780 | 3,223,980,246 | 98.6 | 95.61 | 46.48 | 0.981 | 24,120,224 | 99.45 |
| MC3_72h | 25,295,532 | 3,819,625,332 | 25,180,268 | 3,372,985,384 | 98.42 | 95.23 | 46.27 | 0.837 | 24,797,598 | 98.48 |
| MC1_96h | 25,340,412 | 3,826,402,212 | 25,242,680 | 3,393,141,094 | 98.54 | 95.47 | 46.08 | 0.473 | 24,760,859 | 98.09 |
| MC2_96h | 24,044,604 | 3,630,735,204 | 23,949,816 | 3,216,460,660 | 98.5 | 95.33 | 46 | 0.511 | 23,763,518 | 99.22 |
| MC3_96h | 25,185,662 | 3,803,034,962 | 25,082,934 | 3,327,726,471 | 98.53 | 95.45 | 45.96 | 0.503 | 24,844,323 | 99.05 |

Note: Q20 (%) and Q30 (%) indicate the percentage of total bases for which Phred values greater than 20 and 30 were calculated after quality

Table S2 List of extracellular proteases

| GeneID | Gene | Description | Family | Type | References |
| --- | --- | --- | --- | --- | --- |
| gene0869 | *sspA* | serine protease | S1B | Serine-type endopeptidase EC 3.4.21 | (*1*) |
| gene1565 | *bpr* | bacillopeptidase F | S8A | Serine-type endopeptidase EC 3.4.21 | (*2*) |
| gene1067 | *aprE* | serine protease | S8 | Serine-type endopeptidase EC 3.4.21 | (*2*) |
| gene0646 | *nisP* | serine peptidase | S8 | Serine-type endopeptidase EC 3.4.21 | (*3*) |
| gene1358 | *isp* | major intracellular serine protease | S8 | Serine-type endopeptidase EC 3.4.21 | (*2*, *4*) |
| gene1762 | *aprX* | serine protease | S8 | Serine-type endopeptidase EC 3.4.21 | (*2*) |
| gene3788 | *vpr* | minor extracellular protease | S8 | Serine-type endopeptidase EC 3.4.21 | (*5*) |
| gene3821 | *epr* | minor extracellular protease | S8 | Serine-type endopeptidase EC 3.4.21 | - |
| gene0292 | *pcp* | pyroglutamyl peptidase | C15 | Cysteine endopeptidase EC 3.4.22 | (*2*) |
| gene1510 | *nprE* | metallopeptidase | M4 | Metallo-type endopeptidase EC 3.4.24 | (*2*) |
| gene3324 | *-* | zinc-dependent metalloprotease | M12 | Metallo-type endopeptidase EC 3.4.24 | (*6*) |
| gene3729 | *-* | zinc-dependent protease | M50 | Metallo-type endopeptidase EC 3.4.24 | - |
| gene0840 | *-* | peptidase G2 | G2 | Glutamic-type endopepdase | (*2*) |
| gene1816 | *-* | peptidase G2 | G2 | Glutamic-type endopepdase | (*2*) |
| gene2461 | *pepT* | tripeptidase T | M20B | Aminopeptidase EC 3.4.11-14 | (*2*) |
| gene3865 | *pepT* | tripeptide aminopeptidase | M20B | Aminopeptidase EC 3.4.11-14 | (*2*) |
| gene3386 | *ywaD* | aminopeptidase YwaD | M28 | Aminopeptidase EC 3.4.11-14 | (*7*) |

## References

1. M. R. Sharipova, E. I. Shagimardanova, I. B. Chastukhina, T. R. Shamsutdinov, N. P. Balaban, A. M. Mardanova, G. N. Rudenskaya, I. V. Demidyuk, S. V. Kostrov, The expression of Bacillus intermedius glutamyl endopeptidase gene in *Bacillus subtilis* recombinant strains. *Mol. Biol. Rep.* **34**, 79–87 (2007).

2. Y. Ning, H. Yang, P. Weng, Z. Wu, Zymogram analysis and identification of the extracellular proteases from *Bacillus velezensis* SW5. *Appl. Biochem. Microbiol.* **57**, S27–S37 (2021).

3. A. Kuipers, J. Wierenga, R. Rink, L. D. Kluskens, A. J. M. Driessen, O. P. Kuipers, G. N. Moll, Sec-mediated transport of posttranslationally dehydrated peptides in *Lactococcus lactis*. *Appl. Environ. Microbiol.* **72**, 7626–7633 (2006).

4. H. Yang, Y. Liu, Y. Ning, C. Wang, X. Zhang, P. Weng, Z. Wu, Characterization of an intracellular alkaline serine protease from *Bacillus velezensis* SW5 with fibrinolytic activity. *Curr. Microbiol.* **77**, 1610–1621 (2020).

5. A. Hastuty, W. Mangunwardoyo, M. T. Suhartono, N. Rahmani, P. R. Ferdian, I. Hidayat, Characteristics of thermo-stable serine peptidase vpr from endophytic *Bacillus cereus* strain InaCC-B1657. doi: 10.21203/rs.3.rs-4567703/v1 (2024).

6. V. Cafardi, M. Biagini, M. Martinelli, R. Leuzzi, J. T. Rubino, F. Cantini, N. Norais, M. Scarselli, D. Serruto, M. Unnikrishnan, Identification of a novel zinc metalloprotease through a global analysis of clostridium difficile extracellular proteins. *PLoS ONE* **8**, e81306 (2013).

7. X. Gao, W. Cui, Y. Tian, Z. Zhou, Over‐expression, secretion, biochemical characterisation, and structure analysis of *Bacillus subtilis* aminopeptidase. *J. Sci. Food Agric.* **93**, 2810–2815 (2013).
